# Prospective evaluation of a decision support system providing advice on ventilator settings of: inspiratory oxygen, delivered pressure or volume, frequency and peep

**DOI:** 10.1186/2197-425X-3-S1-A672

**Published:** 2015-10-01

**Authors:** DS Karbing, S Spadaro, SE Rees, CA Volta

**Affiliations:** Aalborg University, Respiratory and Critical Care Group (rcare), Department of Health Science and Technology, Aalborg, Denmark; University of Ferrara, Department of Morphology, Experimental Medicine and Surgery, Section of Anaesthesia and Intensive Care, Arcispedale Sant' Anna, Ferrara, Italy

## Introduction

Management of mechanical ventilation is complex. Guidelines to aid in managing settings may fail to adapt general goals to the individual patient physiology. The Beacon Caresystem (Mermaid Care, Denmark) is a commercial version of a physiological-model based system for advising on mechanical ventilation (1). Mathematical models are tuned to patient measurements allowing advice to be patient specific. Beacon 5 provides advice for control and support modes of ventilation, with advice on: inspiratory oxygen (FiO_2_); inspiratory pressure (PS/PC) or tidal volume (Vt); Positive end-expiratory pressure (PEEP) and, in control modes, respiratory frequency (Rf).

## Objectives

This study investigates short-term changes in ventilator settings and consequent patient status from following advice of the Beacon Caresystem.

## Methods

Thirty patients residing in an ICU in Ferrara, Italy have been included, with a total of 40 patients planned for protocol. Informed consent and ethical approval was obtained in all cases. Advice of the system were followed, if judged appropriate, over a period of 4 hours or until advice was to use current settings. Baseline was routine care. A total of 25 patients were included for analysis with the remainder excluded due to death prior to protocol (1), not meeting inclusion criteria at study start (1) and technical issues with the system (3). Average and spread are reported as mean ± SD or median [25^th^ - 75^th^ perc.] with paired t-test or Wilcoxon's test applied as appropriate for comparing baseline to protocol end.

## Results

Seven and 18 patients were in control and support mode, respectively. Eleven patients (44%) were diagnosed with ARDS. SOFA score and age at day of study were 7.0 ± 2.8 and 67 ± 13 yrs, respectively. Sixteen (64%) of patients were male. Advice was provided 5 ± 2 times. FiO_2_ and PS/PC were reduced from 50 [40-50] to 43 [31-49] % and 12 ± 4 to 9 ± 6 cm H_2_O, respectively (P < 0.05). Vt was reduced from 497 ± 121 to 452 ± 120 ml (7.9 ± 1.9 to 7.2 ± 1.8 ml/kg IBW) (P < 0.05). Rf was increased from 16 [13-21] to 19 [15-24] min^-1^ (P < 0.05). PEEP was not changed significantly with values of 8 [6-9] and 8 [7-10] cm H_2_O at baseline and study end, respectively. Plateau pressure was reduced from 19 ± 5 to 17 ± 7 cm H_2_O (P < 0.05). Pulse oximetry oxygen saturation and end-tidal CO_2_ did not change significantly with baseline to end values of 97 ± 3 to 96 ± 3 % and 5.0 ± 1.1 to 5.1 ± 1.1 %, respectively.

## Conclusions

These initial results indicate that Beacon Caresystem provides rational advice, lowering ventilator support whilst maintaining adequate ventilation and oxygenation.

## Grant Acknowledgment

DSK and SER are minor shareholders and perform consultancy for Mermaid Care.
